# Effects of Charge Trapping at the MoS_2_–SiO_2_ Interface on the Stability of Subthreshold Swing of MoS_2_ Field Effect Transistors

**DOI:** 10.3390/ma13132896

**Published:** 2020-06-28

**Authors:** Xinnan Huang, Yao Yao, Songang Peng, Dayong Zhang, Jingyuan Shi, Zhi Jin

**Affiliations:** 1 High-Frequency High-Voltage Device and Integrated Circuits R&D Center, Institute of Microelectronics, Chinese Academy of Sciences, Beijing 100029, China; huangxinnan@ime.ac.cn (X.H.); yaoyao@ime.ac.cn (Y.Y.); pengsongang@ime.ac.cn (S.P.); zhangdayong1@ime.ac.cn (D.Z.); shijingyuan@ime.ac.cn (J.S.); 2 University of Chinese Academy of Sciences, Beijing 100049, China

**Keywords:** transition metal dichalcogenides (TMDCs), MoS_2_ FET, hysteresis, subthreshold swing, interface states

## Abstract

The stability of the subthreshold swing (SS) is quite important for switch and memory applications in logic circuits. The SS in our MoS_2_ field effect transistor (FET) is enlarged when the gate voltage sweep range expands towards the negative direction. This is quite different from other reported MoS_2_ FETs whose SS is almost constant while varying gate voltage sweep range. This anomalous SS enlargement can be attributed to interface states at the MoS_2_–SiO_2_ interface. Moreover, a deviation of SS from its linear relationship with temperature is found. We relate this deviation to two main reasons, the energetic distribution of interface states and Fermi level shift originated from the thermal activation. Our study may be helpful for the future modification of the MoS_2_ FET that is applied in the low power consumption devices and circuits.

## 1. Introduction

Recently, transition metal dichalcogenides (TMDCs) are leading the trend of studying various two-dimensional (2D) materials. In particular, MoS_2_, which is a representative of the TMDCs, presents an indirect bandgap of 1.29 eV for the bulk form and transforms to direct band semiconductor (bandgap of 1.8 eV) as the number of layers decreases to one [1,2] Additionally, for a top gate MoS_2_ FET, the on/off ratio is up to 10^9^ and the subthreshold swing of is as low as 65 mV/decade, which is comparable to that of its opponent SOI MOSFET, compared to typical values of 10^6^ and close to 80 mV/decade of conventional Si CMOS technology, making MoS_2_ quite suitable for use in switches, logic circuits, amplifiers, and low power dissipation circumstances [3,4,5,6,7].

However, as a 2D material, because of the extremely high surface-to-volume ratio, the properties of MoS_2_ FETs, such as threshold voltage, hysteresis, on/off ratio, and *SS*, can be easily affected by interface states at the interface between the MoS_2_ channel and the gate dielectric [5,8]. During the sweep of gate voltage, the interface states are electrically equivalent to an additional capacitance which is first in parallel to the semiconductor capacitance, and their result is then series to the oxide capacitance by exchanging electrons with the MoS_2_ channel, thus sabotaging the stability of MoS_2_ FETs. Among the device performance stability issues caused by the interface states, the enlargement of *SS* will slow down the switch speed and hugely increase the static power consumption of FETs, which will hinder the use of MoS_2_ FETs in low-power applications [9,10].

*SS* is normally considered to have a linear relationship with temperature. This might be valid for top gate FETs whose gate dielectric capacitance is large enough so that the influence of interface states capacitance (*C_it_*) could be ignored. However, for research concerns, a back gate metal-oxide-semiconductor (MOS) structure based on SiO_2_ dielectric is usually adopted to study the basic properties of MoS_2_ FETs whose *SS* is typically more than 1000 mV/dec [11]. Note that the *SS* can be hugely decreased by replacing SiO_2_ with a high-k dielectric [12].

Thus, for the first time, an obvious deviation of *SS* from its linearity with temperature is observed for the backgated MoS_2_ FET with 300 nm SiO_2_ dielectric. We explain this anomalous phenomenon with the energetic distribution of interface states density and the temperature induced Fermi level shift. According to these results, *SS* stability of the devices critically relies on a rational design of gate dielectric.

## 2. Experiments

**Figure 1:**
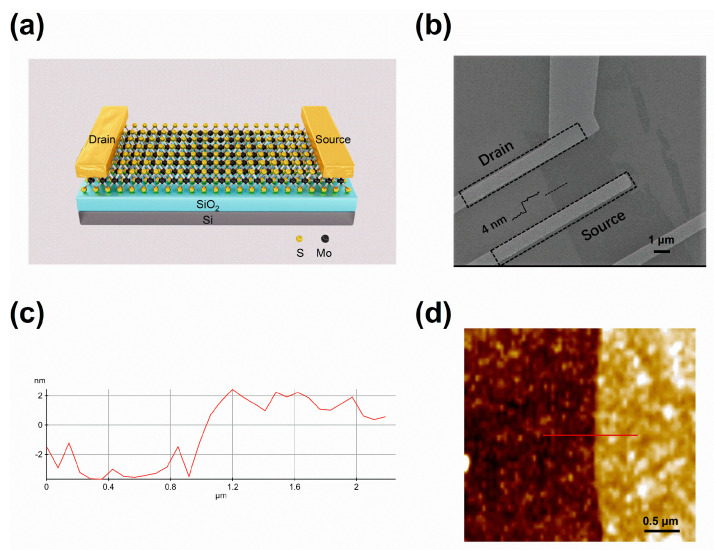
(**a**) Schematic of the fabricated MoS_2_ FET. (**b**) SEM image of the MoS_2_ FET. The drain and sources are indicated by dashed boxes. (**c**,**d**) AFM profile revealing the MoS_2_ thickness along the red line.

In our study, with the scotch tape method [13,14], the device was prepared by mechanically exfoliating MoS_2_ thin films onto 300 nm SiO_2_ grown on low resistive Si substrate. Drain and source electrode pads of Ti/Au (10/100 nm) were formed through e-beam lithography, e-beam evaporation and lift-off processes in sequence. Figure 1a,b show the schematic view and colored SEM (Tescan, Brno, Czechoslovakia) image of the fabricated MoS_2_ FET. The channel length and width of the device were about 3 µm and 5 µm, respectively. The thickness of the MoS_2_ film was about 4 nm measured using a Park Systems’ atomic force microscope (AFM, Park Systems, Hyderabad, India) as shown in Figure 1c,d. The MoS_2_ FET was placed in a shielded probe station under vacuum condition (less than 10^−5^ mbar) in order to minimize the influence of water and oxide molecules in ambient air. All electrical transport characteristics were measured using an Agilent B1500 semiconductor parameter analyzer (Agilent, Santa Clara, CA, US) under dark conditions. The transfer characteristics were obtained by sweeping *V*_BG_ from a negative gate voltage *V_BG, min_* to a positive gate voltage *V_BG, max_* (forward sweep), and then back to *V_BG, min_* (backward sweep) again at a constant *V_DS_* = 1 V at room temperature. Wherein, *V_BG, max_* was fixed at 50 V and *V_BG, min_* was varied from −50 V to −80 V in steps of −10 V for each measurement, respectively.

## 3. Results and Discussions

**Figure 2:**
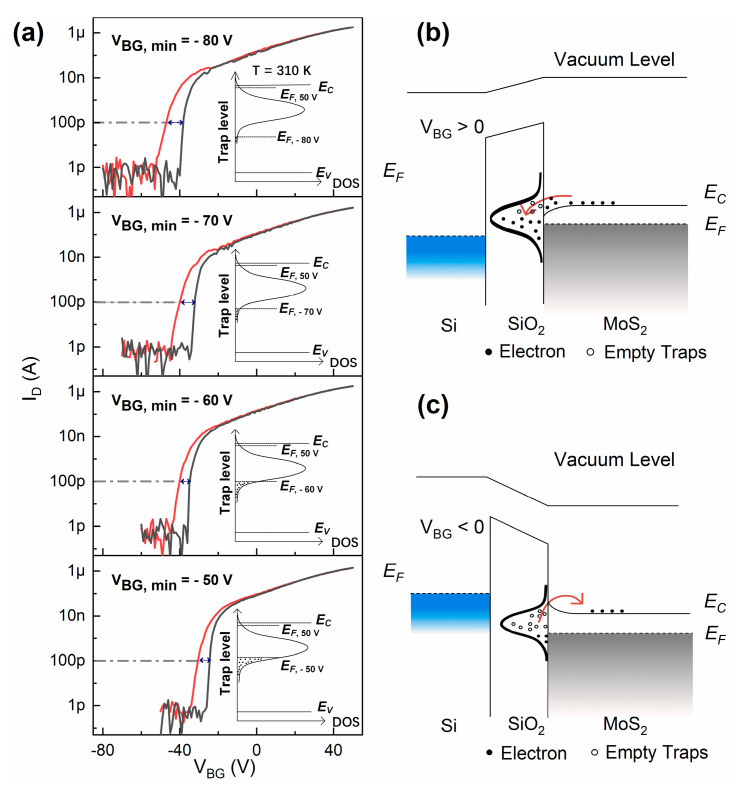
(**a**) The transfer curves for forward sweep (red line) and backward sweep (black line) measured at 310 K. The hysteresis is selected as the voltage shift between the transfer curves of the forward sweep and the backward sweep at *I*_DS_ = 100 pA, which is indicated by a double-headed arrow. The inset shows the positions of the Fermi level corresponding to *V_BG, min_*s of −50 to −80 V, respectively. (**b**,**c**) The band structure of MoS_2_ channel considering the interface states at the MoS_2_–SiO_2_ interface.

Figure 2a represents the transfer curves of the MoS_2_ FET measured at 310 K with varying *V_BG, min_*s. With *V_BG, min_* varied from −50 V to −80 V, the threshold voltage (*V_TH_*) of the device negatively shifted for both forward sweep and backward sweep. For forward sweeps, the threshold voltage of the device shifted from −30 to −43 V. For backward sweeps, the threshold voltage of the device shifted from −23 to −35 V. Meanwhile, the hysteresis of the device increased from 5 to 9 V. For MoS_2_ FETs, threshold voltage shift and hysteresis are mainly caused by the interface states acting as charge traps at the MoS_2_–SiO_2_ interface. These interface states are the result of dangling bonds formed at the surface of SiO_2_ during the growth process of SiO_2_. By exchanging electrons with the MoS_2_ channel, the interface states can be viewed as a “impurity band” in the MoS_2_ bandgap [15,16]. At 310 K, as the gate voltage sweeps from *V_BG, min_* to 50 V, the conduction band bends downward relative to the Fermi level. The positions of the Fermi level corresponding to *V_BG, min_*s of −50 to −80 V are shown, respectively, in the inset of Figure 2a. As the gate voltage increases, a part of energy levels of the interface states will be swept over by the Fermi level. This will cause the charge traps to capture/release electrons. When the energy levels of the interface states are below the Fermi level, it indicates the energy level are occupied by electrons as shown in Figure 2b. When the energy levels of the interface states are above the Fermi level, electrons that used to be trapped in these interface states tend to get de-trapped, as shown in Figure 2c. When a negative gate bias is applied at the beginning of forward sweep, a portion of electrons are released to the MoS_2_ channel, the empty traps become positively charged. This will weaken the depletion of the MoS_2_ channel, because the positive charges will partially “screen” the negative gate voltage. The threshold voltage will then negatively shift. For a more negative *V_BG, min_*, more positive charges will be present. Then, it will require a more negative gate bias to depletion the MoS_2_ channel. Therefore, the threshold voltage will keep on shifting to the negative direction. As for the hysteresis, because we used a constant gate voltage sweeping rate of 2 V/s, the accumulation time interval in one gate voltage sweep cycle is 50 s. The depletion time interval from *V_BG, min_* to 0 V in the forward sweep is shorter than the accumulation time interval, even for the *V_BG, min_* of −80 V condition. Together with the fact that the de-trapping process is much lower than electron movements in the MoS_2_ channel [8], the refilled traps during the accumulation process will partly screen the negative gate voltage and cause a positive shift of *V_TH_*. Thus, the hysteresis occurs.

Like hysteresis and threshold voltage shift, *SS* shift can also be attributed to the interface states. However, the *SS* shift can hardly be observed without gate bias stressing [17], because the density of states (DOS) distribution of interface states is seldom considered. In this study, DOS distribution of the interface states is assumed to coincide with Gaussian distribution and its maximum value is located at a few tens of meV below the conduction band, as shown in Figure 2 [15,18,19]. For both forward sweeps and backward sweeps, as the gate voltage sweeping range expands, the *SS* of the device is enlarged because more traps are depleted or in other words “activated” when larger negative gate bias is used. *SS* increases from 2188 to 2898 mV/dec for forward sweeps. *SS* increases from 648 to 1550 mV/dec for backward sweeps. For forward sweeps, the interface states density (*D_it_*) is extracted to be 2.64 × 10^12^ eV^−1^cm^−2^ for *V_BG, min_* of −50 V, and *D_it_* is extracted to be 3.49 × 10^12^ eV^−1^cm^−2^ for *V_BG, min_* of −80 V, by using the following equations [20]:

(1) $$SS=\ln10\times kT/q\times \left(1+C_{it}/C_{OX}\right)$$

(2) $$D_{it}=C_{it}/q^{2}$$

where *k* is the Boltzmann’s constant, *T* is the measurement temperature, *q* is the electron charge, and *C_ox_* is the gate dielectric capacitance density which is 11.5 nF/cm^2^ (*C_ox_* = *ɛ*_0_*ɛ*_r_/d; *ɛ*_r_ = 3.9; *d* = 300 nm) for the MoS_2_ FET.

**Figure 3:**
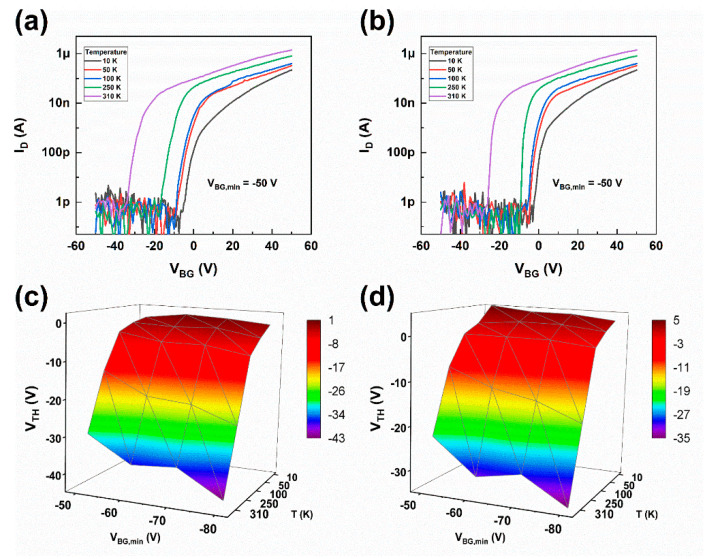
Transfer curves at a series of temperatures (10–310 K) for forward sweep (**a**) and backward sweep (**b**), respectively. Relationship between *V_TH_*, *V_BG, min_*, and temperature for forward sweep (**c**) and backward sweep (**d**), respectively.

To further study the mechanism of the *SS* shift, we conducted transfer characteristics measurements at a series of temperatures. Figure 3a,b show the transfer curves for *V_BG, min_*s of −50 V for forward and backward sweeps, respectively, in a temperature range from 10 to 310 K. The *V_TH_* shifts negatively as temperature increases. The transfer curves for *V_BG, min_*s from −60 to −80 V in steps of −10 V with constant *V_DS_* = 1 V were also measured. The relationship between *V_TH_*, temperature, and *V_BG, min_* is extracted and illustrated in Figure 3c,d for forward and backward sweeps, respectively. For more negative *V_BG, min_*s, the *V_TH_* shift enlarges more abruptly compared to less negative *V_BG, min_*s when the temperature is above 100 K, which means the “screening” mentioned above becomes stronger above 100 K. No gate bias stressing is applied in this experiment, therefore the electron exchange between the MoS_2_ channel and interface states is incomplete. For more complete delegation by larger gate bias, the “effective” *D_it_* is higher. As the unoccupied traps are positively charged, the electrons in the MoS_2_ channel will suffer from Coulomb scattering. We have $\sigma_{DS}\propto n^{\alpha }$, wherein $\sigma_{DS}$ the conductance of the MoS_2_, 1≤α≤2 is the coefficient that reflects the screening of Coulomb scattering. If the Coulomb scattering is fully screened, then α=1. For bare Coulomb scattering, α=2. As $I_{DS}\propto \sigma_{DS}$, $V_{BG}-V_{TH}\propto n$, the coefficient α is the slope of $\ln I_{DS}$ versus $\ln\left(V_{BG}-V_{TH}\right)$ [8]. By calculating the data from Figure 3a,b, we obtain a minimum α of 1.56 for *V_BG, min_* of −80 V and a maximum α of 1.72 for *V_BG, min_* of −50 V at 310 K. The fact that more negative *V_BG, min_* corresponds to weaker Coulomb scattering indicates the extra electrons depleted to the MoS_2_ channel by the larger *V_BG, m_* (more negative) is more effective on screening the Coulomb scattering caused by the unoccupied traps than causing more severe scattering. This also suggests large amount of interface states intrinsically exist at the MoS_2_/SiO_2_ interface.

**Figure 4:**
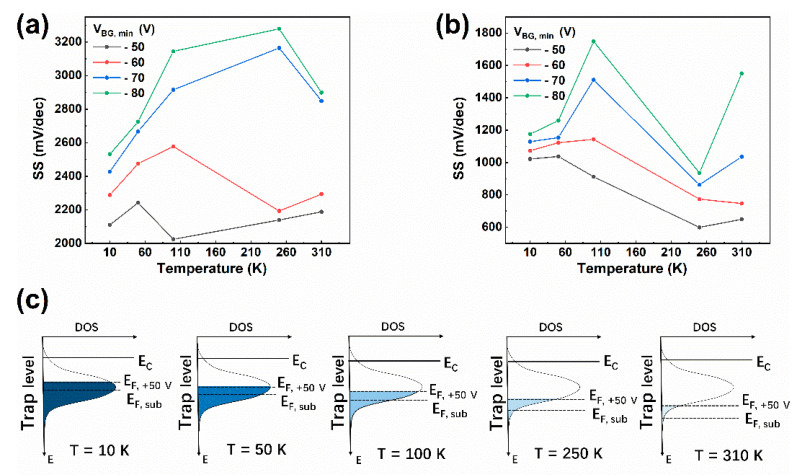
Relationship between *SS* and temperature for forward sweep (**a**) and backward sweep (**b**), respectively. (**c**) Schematic of Fermi level shifting progress of MoS_2_ channel relative to the interface states and conduction band caused by the increasing of temperature from 10 to 310 K for *V_BG, min_* of −50 V. *E_F, +50 V_* and *E_F, sub_* correspond to the Fermi level for gate voltage of +50 V and subthreshold voltage, respectively.

Figure 4a,b show the relationship between *SS* and temperature extracted from Figure 3a,b for forward sweep and backward sweep, respectively. Normally, *SS* should have a linear relationship with temperature. However, for forward sweeps, for *V_BG, min_* of −50 V, the *SS* versus *T* curve increased from 10 K and reached a maximum mV/dec at 50 K, then dropped to a minimum at 100 K, and began to increase linearly above 100 K. The *SS* versus temperature curve presents an abnormal bulge in the temperature range of 10 to 100 K. For *V_BG, min_* of −60 V, the bulge expands to 250 K and *SS* begins to increase linearly from there on. As *V*_BG, min_ further decreases to −70 V, the bulge expands at least to 310 K. For *V_BG, min_* of −80 V, the range of the bulge is similar to that for *V_BG, min_* of −70 V. Although for *V_BG, min_*s of −70 and −80 V, the *SS* does not show a linear increase in the temperature range of this experiment; we believe the *SS* will still begin to increase linearly at a higher temperature. For backward sweeps, the range of the bulge is almost the same for each *V_BG, min_*. The *SS* curves all begin to increase linearly at around 250 K. Moreover, the scale of the bulge is even larger for small *V_BG, min_*s compared to forward sweeps. For this abnormal deviation of *SS* from linearity with temperature, the energetic distribution of interface states density and the temperature induced Fermi level shift mechanisms are adopted to explain it.

Due to the trapping and releasing processes of electrons in the traps, the interface states can be viewed as a capacitor. The capacitance *C_it_* is determined by the density of the interface states *D_it_*. Thus, *D_it_* is the key to study this abnormal relationship between *SS* and temperature. As we assume *D_it_* has Gaussian distribution as shown Figure 2, it can be written as

(3) $$D_{it}\left(E\right)=D_{it,\;max}\left(E\right)\times f\left(E\right)$$

and *f* (*E*) in Equation (3) can be written as

(4) $$f\left(E\right)=\exp\left[-4\times \log2\times {\left(\frac{E_{F}\left(T\right)-E_{D}}{\mathrm{FWHM}}\right)}^{2}\right]$$

where in *D_it, max_* (*E*) is the maximum value the *D_it_* can reach during the shift of Fermi level as temperature increases, *E_F_* (*T*) is the location of Fermi level as a function of temperature, and *f* (*E*) is the energetic distribution function of the interface states [19,21]. *f* (*E*) represents the possibility that a certain energy level is occupied. *FWHM* is the full width at half maximum of *D_it_*. On the other hand, because of the activation of bulk charge traps (including bulk MoS_2_ trap charges, fixed oxide charges, and oxide trap charges inside a thick SiO_2_ insulator) and interface states [22], the location of the Fermi level can be modulated by temperature and can be expressed as

(5) $$E_{F}=(E_{C}/E_{D})/2+\left(kT/2\right)\ln\left(N_{D}/2N_{C}\right)$$

(6) $$E_{F}=E_{C}+kT\ln\left(N_{D}/N_{C}\right)$$

corresponding to low temperature weak ionization region (Equation (5)) and high temperature strong ionization region (Equation (6)), wherein *E_C_*, *N_C_*, and *N_D_* are the bottom of the conduction band, effective density of states of conduction band, and donor concentration of the interface states for MoS_2_, respectively. As shown in Figure 4c, the position of the Fermi level corresponding to subthreshold situation (*E_F, sub_*) shifts downward relative to the energy levels of the interface states when temperature increases from 10 to 310 K. Thus, the *D_it_* corresponding to *E_F, sub_* will change with temperature.

Due to the Gaussian distribution, *D_it_* has a maximum value *D_it, max_*. For a certain temperature, the more the Fermi level approaches *D_it, max_*, the larger the *SS* is and vice versa. When substituting above Equations (2)–(6) back into Equation (1), the expression of *SS* can be written as

(7) $$SS=q\ln10\times kT\times D_{it,\;max}\left(E\right)\times \exp\left[-4\times \log2\times {\left(\frac{E_{F}\left(T\right)-E_{D}}{\mathrm{FWHM}}\right)}^{2}\right]/C_{OX}+kT/q\times \ln10$$

The deduced expression for *SS* has two terms. The first term is a nonlinear term which corresponds to the depletion of interface states by the gate voltage, and the second term is a linear term which is only affected by temperature. Taking the situation for *V_BG, min_* of −50 V as an example, for forward sweep, from 10 to 50 K, *D_it_* corresponding to *E_F, sub_* increases rapidly. Thus, the nonlinear term of Equation (7) dominates and the *SS* curve begins to increase and deviate from linearity. After 50 K, as *E_F, sub_* passed *D_it, max_*, the nonlinear term begins to decrease. If the increasing of the linear term and the decreasing the nonlinear term reach an equilibrium, the *SS* will saturate. When temperature continues to increase, the drop down of *D_it_* will experience an acceleration. The nonlinear term will dominate again during this fast drop and *SS* will drop with it. When the decrease of *D_it_* slows down at a position that is far from *D_it, max_*, another equilibrium with the linear term will be accomplished. Thus, *SS* will reach a minimum. As the temperature further increases, the linear term will dominate and the nonlinear term will be negligible. The *SS* will increase linearly along with the linear term. When *E_F, sub_* shifts downward as temperature increases, because the effective *D_it_* is actually higher compared to the situation for *V_BG, min_* of −50 V, the *SS* will be generally larger, as can be seen in Figure 4a,b and the shift of *E_F, sub_* will be slowed down in the temperature dimension because more traps have to be thermally activated. Then the maximum or minimum of *SS* will be achieved at higher temperatures. The above discussion also explained why the deviation from linearity is hardly observed for small gate voltage sweep ranges (e.g., from −30 V to 30 V) in which case the effect of the nonlinear term is too small and is covered up by the linear term. For backward sweep, because the traps refilled when the gate voltage is positive have not fully de-trapped yet. Then, effective *D_it_* becomes smaller compared to forward sweep. The *SS* for backward sweep is thus generally smaller than that for forward sweep. This result coincides with the fact that the *V_TH_* shift for backward sweep is generally smaller than that for forward sweep, which also originates from the more incomplete depletion of interface states for backward sweep. From the above discussion, we can infer that the *FWHM* of *D_it_* is important for whether the deviation of *SS* from linearity can be observed. If the *FWHM* is small enough, the distribution of *D_it_* can be viewed as a step function. The slope of *SS* will change abruptly to another value at a certain temperature as is observed by Park et al. [22,23]. However, if the *FWHM* is large, the situation will become what we observed for our MoS_2_ FET.

## 4. Conclusions

In conclusion, we have investigated the *SS* instability with temperature of back-gated multilayer FET device induced by interface states in the temperature range of 10–310 K. We found that the relationship between *SS* and temperature will deviate from linearity if the energetic distribution of the interface states is abroad enough. Because of the Fermi level shift with temperature induced by the terminal activation of bulk charge traps and interface states, and broad interface states density distribution, the capacitor effect of the interface states can greatly influence the *SS* instability with temperature. Moreover, we can potentially predict the relationship between *SS* and temperature for MoS_2_ FETs with other interface states situations. Our study is helpful for understanding interface properties of MoS_2_ FETs and has important implications for gate dielectric material modification for low power applications based on MoS_2_.
